# Evolutionary dynamics of the multidrug-resistant Salmonella Infantis harbouring the pESI megaplasmid across Europe

**DOI:** 10.1099/mgen.0.001787

**Published:** 2026-07-13

**Authors:** Patricia Alba, Elena Lavinia Diaconu, Virginia Carfora, Antonio Battisti, Alessia Franco, Pieter-Jan Ceyssens, Cristina Graells-Garcia, Jannice Schau Slettemeås, Magdalena Zając, Magdalena Skarżyńska, Dariusz Wasyl, Lurdes Clemente, Manuela Caniça, Vera Manageiro, Stefan Börjesson, Eelco Franz, Emma Östlund, Robert Söderlund, Aurora Garcia-Fernandez, Laura Villa

**Affiliations:** 1 Department of General Diagnostics, National Reference Laboratory for Antimicrobial Resistance, Istituto Zooprofilattico Sperimentale del Lazio e della Toscana “M. Aleandri”, Rome, Italy; 2 Sciensano, Department of Human Infectious Diseases, Brussels, Belgium; 3 Norwegian Veterinary Institute, Department of Animal Health, Welfare and Food Safety, Ås, Norway; 4 Department of Bacteriology and Bacterial Animal Diseases, National Veterinary Research Institute (PIWET), Al. Partyzantów 57, 24-100 Puławy, Poland; 5 Laboratory of Bacteriology and Mycology, National Reference Laboratory of Animal Health, INIAV-National Institute of Agrarian and Veterinary Research, Oeiras, Portugal; 6 National Reference Laboratory of Antibiotic Resistances and Healthcare Associated Infections, Department of Infectious Diseases, National Institute of Health Doctor Ricardo Jorge, Lisbon, Portugal; 7 Public health Agency of Sweden, Department of Microbiology, Solna, Sweden; 8 National Institute for Public Health and the Environment (RIVM), Centre for Infectious Disease Control, Bilthoven, Netherlands; 9 Swedish Veterinary Agency, Department of Microbiology, Uppsala, Sweden; 10 Swedish University of Agricultural Sciences, Department of Clinical Sciences, Uppsala, Sweden; 11 Department of Infectious Diseases, Istituto Superiore di Sanità, 00161 Rome, Italy

**Keywords:** extended-spectrum beta-lactamase (ESBL), multi-drug resistance, One Health, pESI plasmid, *Salmonella* Infantis, whole-genome sequence

## Abstract

Multidrug-resistant *Salmonella enterica* subsp. *enterica* serovar Infantis clone, harbouring the pESI megaplasmid, first described in Israel in 2014, is consistently reported in poultry and humans worldwide. This study aimed to investigate the genomic epidemiology of *S*. Infantis collected by nine European Public Health Institutions from samples of different origins (human, food and animal sources) and understand the evolutionary dynamics of pESI-like in Europe. The resolved pESI-like sequences have also been compared with complete publicly available pESI-like sequences from other countries, in a One Health context. The circulation of a *S*. Infantis clone in Europe carrying the mosaic megaplasmid pESI-like has been associated with resistance to sulphonamides (*sul*)*,* tetracycline (*tet*)*,* streptomycin and spectinomycin (*aadA1*). In recent years, *bla*_CTX-M-1_-positive pESI-like plasmids have been increasingly detected in the extended-spectrum beta-lactamase-producing *S*. Infantis clone. Using a combined short- and long-read sequencing approach, two main types of pESI variants have been identified, differing in the accessory gene content, including the *bla*_CTX-M_ variant, indicating a certain stability of pESI variants detected in different geographical regions and sources over time (2011–2021). Moreover, the differences were related to the acquisition of resistance, virulence or fitness-enhancing genes that would potentially benefit the *Salmonella* host.

**Impact Statement**

Multidrug-resistant *Salmonella enterica* serovar Infantis is a major public health concern in Europe, where it is a leading cause of human salmonellosis and highly prevalent in poultry. This study advances current knowledge by providing a comprehensive genomic analysis of *S*. Infantis and its associated pESI-like megaplasmid across human, food and animal sectors in Europe, using a combined short- and long-read sequencing approach. By resolving complete pESI-like plasmid structures, we demonstrate that two main pESI variants have circulated stably across countries, sources and over more than a decade, while accumulating distinct resistance, virulence and fitness-associated genes, including different *bla*_CTX-M_ variants. The findings are of broad relevance to public health authorities, food safety agencies and antimicrobial resistance surveillance programmes, as they highlight the central role of pESI variants in the persistence and success of multidrug-resistant *S*. Infantis within a One Health framework.

## Data Summary

The raw sequences, short and long, generated during this project are available in European Nucleotide Archive (ENA) under the study accession number PRJEB96843; the accession numbers list is provided in the supplementary table. All supporting data, code and protocols have been provided within the article or through supplementary data files.

## Introduction

*Salmonella* Infantis is in the EU the fourth most common serovar reported in clinical cases of human salmonellosis and is the first and third serovar reported in broilers and turkeys, respectively [1]. An essential trait of *S*. Infantis is its multidrug-resistant (MDR) phenotype, including resistance to the critically important third-generation cephalosporins, quinolones, sulphonamides (or sulphamethoxazole-trimethoprim) and tetracyclines [2]. In 2022, *S*. Infantis isolates contributed most to MDR levels in all poultry populations, 74.4% for broilers, 32.4% for laying hens and 31.5% for turkeys. In humans, MDR *S*. Infantis isolates were also reported at high levels, 42.4% in 2023 [2].

The high reported resistance levels to certain antimicrobial classes align, at least partially, with the circulation of a *S*. Infantis clone in Europe carrying the mosaic megaplasmid pESI-like (plasmid for emerging *S*. Infantis). The megaplasmid pESI was first described in Israel in 2014 and carried genes encoding sulphonamides (*sul*), tetracycline (*tet*), streptomycin and spectinomycin (*aadA1*) resistance [3]. An MDR extended-spectrum beta-lactamase (ESBL)-producing *S*. Infantis clone, harbouring a pESI-like megaplasmid carrying *bla*_CTX-M-1_, was subsequently described in food-producing animals, meats thereof (poultry) and humans in Italy [4,5]. The same plasmids were later found in other European countries such as the Netherlands, Germany and the UK [6,7]. These pESI-like plasmids maintain the mosaic structure previously described, with the following scaffold regions: (i) an IncFIB replicon type (pN55391 plasmid); (ii) a non-functional IncP-1 alpha origin of replication; (iii) strong conservation of the IncI1 reference plasmid R64 backbone, as *ardA*, *pilL*, *sogS* and *trbA* genes, except the region encoding the IncI1 replication origin; (iv) different toxin/antitoxin systems; (v) the yersiniabactin operon with 97.4% similarity with the yersiniabactin operon of *Yersinia pestis* (AF091251); (vi) pESI_backbone_gene; (vii) K88-like fimbria; and (viii) a second Infantis plasmid-encoded fimbria (*fim*) [3,6]. It also contains the following variable regions: (i) two integrons, one containing the integrase *intI1*, mainly associated with the ESBL gene *bla*_CTX-M-1_ and *dfrA1*, *sul1* and *qa*cΔE1 genes (conferring resistance to trimethoprim, sulphamethoxazole and disinfectants, respectively) and one containing the integrase *intI2* associated with the trimethoprim-resistance gene variant *dfrA14*, (ii) the complete mercury resistance (*mer*) operon, (iii) the tetracycline resistance gene *tet*(A) and (iv) the *impABC* operon associated with resistance to UV irradiation [8].

In the Italian broiler chicken industry, the MDR *S*. Infantis clone producing CTX-M-1 is still prevalent [1], and since 2015, it has been transmitted along the food chain to humans [4]. A previous European study on the molecular epidemiology of MDR *S*. Infantis, based on short-read whole-genome sequencing (WGS), found no correlation between chromosomal variation and source or geographical location, but the pESI-like plasmids were more homogeneous [6].

In the USA, another *S*. Infantis clone harbouring the pESI-like plasmid carrying the ESBL gene *bla*_CTX-M-65_ has been described in food-producing animals and food [9]. This clone was later found among European human clinical isolates and was associated with a travel to Asia and South America [4,6, 7, 10, 11]. However, 14 likely domestically acquired cases of *S*. Infantis with *bla*_CTX-M-65_ were reported in Denmark, Germany, Spain and the Netherlands between 2019 and 2021, although there was no evidence that the CTX-M-65-producing *S*. Infantis clone had entered the food-producing animal industry in Europe [6,12]. Similarly, to the CTX-M-1-producing *S*. Infantis, most of the CTX-M-65-producing isolates were resistant to ciprofloxacin, nalidixic acid, sulphamethoxazole, tetracycline and trimethoprim [12]. However, resistance to phenicols, fosfomycin and aminoglycosides was mainly due to the presence of other resistance genes, *floR*, *fosA* and *aph*(4)-Ia. Recently, another pESI-like plasmid, harbouring the *bla*_CTX-M-14_ gene, was described in Russia [13]. pESI-like plasmids have also been described in other *Salmonella* serovars, such as *S*. Muenchen [14], *S*. Senftenberg and *S*. Alachua [14,15]. In addition, *S*. Agona and *S*. Schwarzengrund have also been found to harbour the pESI core genes [14].

The objective of this study was to analyse the genomic epidemiology of *S*. Infantis in human, food and animal sectors and understand the evolutionary dynamics of pESI-like plasmids in Europe, using a combined short- and long-read sequencing approach to study SNPs and structural variations of the plasmid. From the One Health perspective, we characterized the evolution of the pESI variants of *S*. Infantis, which, as far as we know, is not present in Europe.

## Methods

### Sample collection of *S*. Infantis

**Fig. 1.**
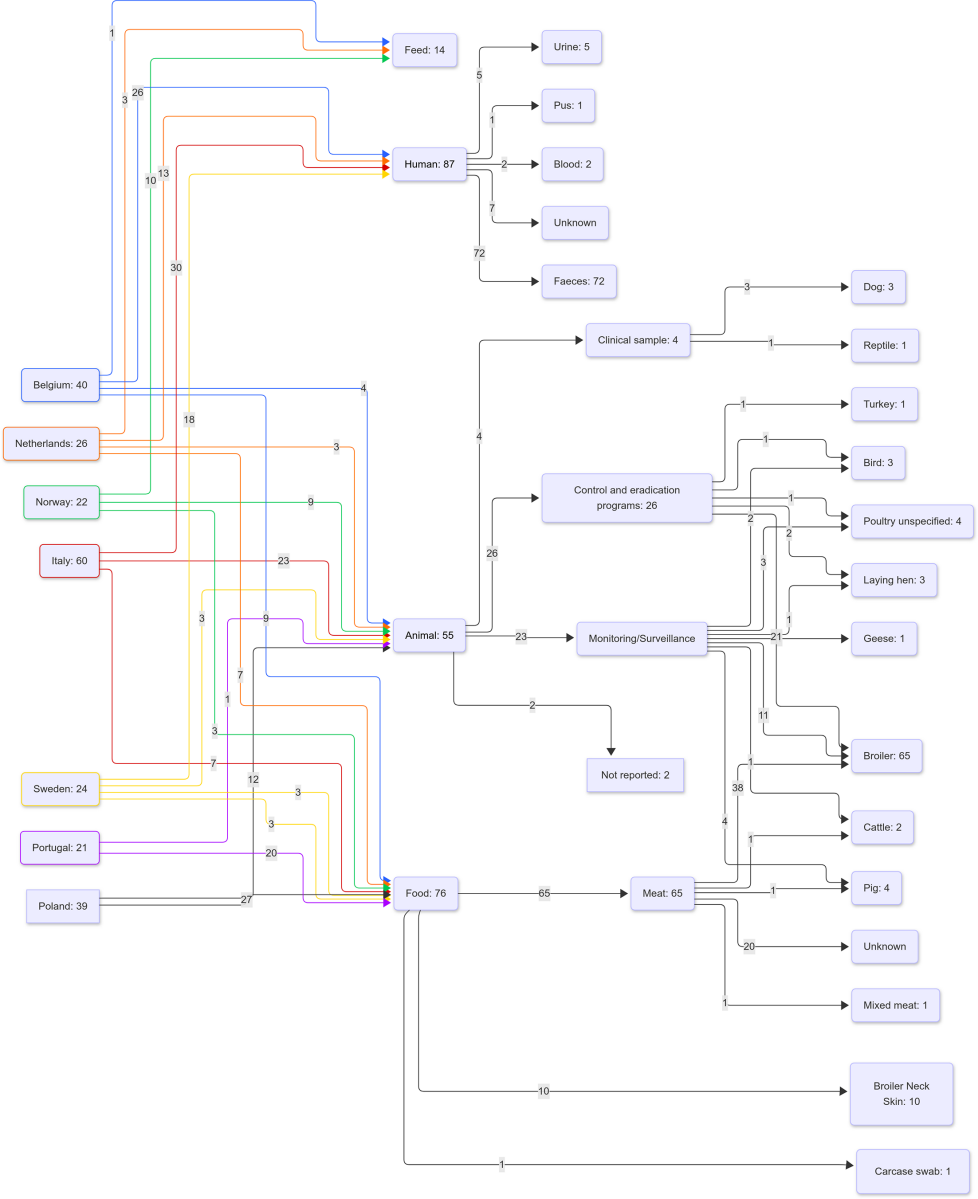
Flowchart indicating the country, source and matrix of the isolates used in this study.

A total of 232 WGS short reads of *S*. Infantis isolates from different sources and years of isolation (from 1999 to 2020) were collected by nine European public health institutions and veterinary institutions, as part of the partners of the One Health European Joint Programme project Full-Force (https://onehealthejp.eu/projects/antimicrobial-resistance/jrp-full-force) (accessed on 11 June 2025) (Fig. 1). The selection of isolates was based on the presence of typical markers of the pESI-like plasmid, or on phenotypic or genotypic tetracycline and sulphonamide resistance profiles. Phenotypic data on antimicrobial susceptibility and/or the presence of IncFIB(pN55391), *tet*(A) and *sul*1 genes in the isolates were already available from each participating country. The *S*. Infantis isolates were collected from Italy (*n*=60), Belgium (*n*=40), Poland (*n*=39), the Netherlands (*n*=26), Sweden (*n*=24), Norway (*n*=22) and Portugal (*n*=21). Of these, 87 isolates were of human origin, 76 from food (meat from poultry, pigs, cattle or meat preparations), 55 from animals and 14 from feed (Table S1, available in the online Supplementary Material, Fig. 1).

Of 55 animal isolates, 46 were from food-producing animals: 41 from poultry (broilers, laying hens, goose, turkey or unspecified poultry), 4 from pigs and 1 from cattle. The remaining nine isolates were from three dogs, two wild birds, two reptiles, one quail and one wild red fox. The isolates were collected from various types of matrices and from different sampling contexts (Suppl. Table, Fig. 1): control and eradication programmes (*n*=45), active or passive monitoring programmes (*n*=39), surveillance (*n*=5), surveys (*n*=4), clinical cases (*n*=5) and unspecified/unknown (*n*=5). For 129 isolates, the sampling context was not reported. Out of 87 isolates of human origin, 72 were from faeces, five from urine, two from blood, one from pus and seven were of unknown sample type (Suppl. Table; Fig. 1).

### Bacterial short- and long-read sequencing and hybrid assembly

**Fig. 2.**
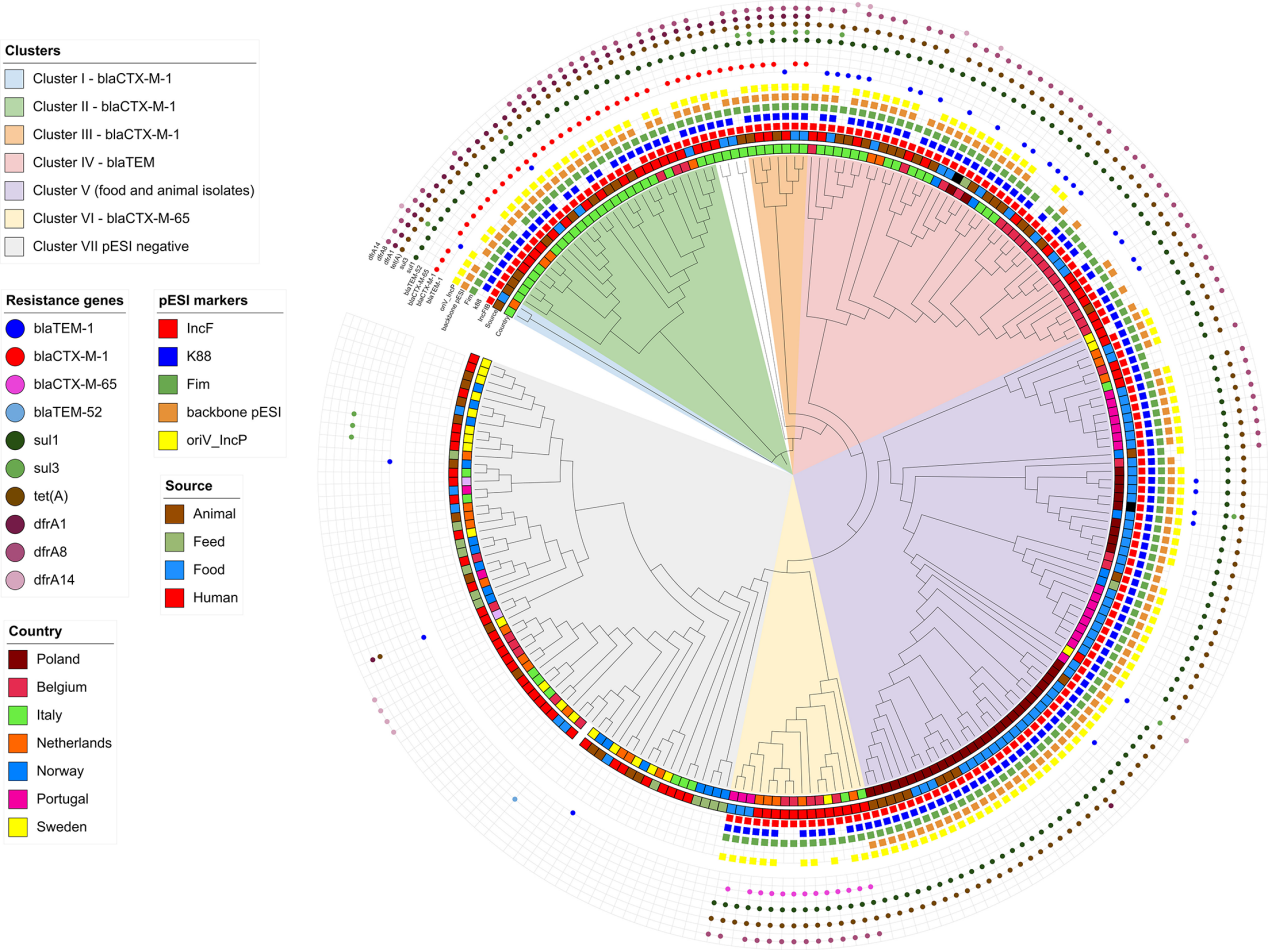
SNP tree of the 232 *S*. Infantis collected without branch length. Metadata associated with the isolates, from inner to outside circle: country, source, pESI backbone markers (IncFIB, *k88*, *Fim*, backbone pESI_ backbone_gene and oriV_IncP) and resistance genes (*bla*_TEM-1_, *bla*_CTX-M-1_, *bla*_CTX-M-65_, *bla*_TEM-52_, *sul1*, *sul3*, *tet*(A), *dfrA1*, *dfrA8* and *dfrA14*).

Illumina short reads were trimmed using TrimmomaticPE v0.22 [16] and assembled using Spades v3.13 [17] with the ‘--careful’ parameter. The serotype was confirmed using SISTR (sistr_cmd 1.1.1; Yoshida et al., 2016) and multilocus sequence typing (MLST) was performed using the MLST tool (https://github.com/tseemann/mlst) and the Achtman scheme [18]. Assemblies were additionally compared by SNP analysis, using Snippy v4.4.3 (https://github.com/tseemann/snippy), with LN649235.1 as a reference. In parallel, a Mash analysis using mashtree v1.2.0 [19] was carried out. The resistome and plasmidome of each isolate were obtained with ABRicate v1.0.1 (https://github.com/tseemann/abricate) using ResFinder (9 January 2026) [20], AMRFinder (v4.2.7, last update 21 January 2026) and PlasmidFinder [21] databases, using 95% as the threshold for coverage and identity. The presence of the following described markers for pESI-like plasmids was tested using ABRicate v1.0.1, with a custom database of pESI backbone region that includes different pESI backbone markers: pESI_backbone_gene, K88, Fim, oriV_IncP1alpha plasmid, DNA replicase and *qac*ΔE1 (Table S1) [3,4]. Forty-eight *S*. Infantis isolates from the seven European partners (human, animal, food and feed sources) were selected for Oxford Nanopore Technology (ONT) sequencing based on the representativeness of human, animal, food and feed isolates in the various clusters identified in the genomic clustering obtained from short-read data analysis (Fig. 2). Libraries were prepared with the rapid barcoding kit (SQK-RBK004) and sequenced by each partner using R9.4.1 flow cells on the nanopore-based MinION device [22]. Basecalling was performed with the high-accuracy algorithm.

Long reads were analysed using an in-house pipeline developed at the Italian National Reference Laboratory for AMR (NRL-AR), IZSLT, based on the Full-Force Plasmid Assembler (https://github.com/MBHallgren/FullForcePlasmidAssembler). ONT reads were trimmed with Trimmomatic v0.39 (https://github.com/usadellab/Trimmomatic), and quality was assessed with FastQC v0.11.9 (GitHub - s-andrews/FastQC). Then, ONT reads were filtered by quality using NanoFilt v2.8.0 (-q 10 --headcrop 75l 500) [23]. Unicycler v0.4.7 (https://github.com/rrwick/Unicycler) was used for hybrid assembly from the trimmed and filtered ONT reads and the trimmed Illumina reads. Resulting assemblies were polished using Pilon v1.24, using BWA v0.7.17 as an aligner [24].

### Plasmid pairwise distance, annotation and pangenome analysis

To estimate the pairwise distance between the pESI-like complete sequences, a Mash triangle approach based on the MinHash algorithm [19] was used, and the results were represented as a multidimensional scaling (MDS) plot using Plotly v4.10.0 (https://github.com/plotly/plotly.R). The gene-by-gene analysis was performed on fully resolved pESI-like plasmid sequences. After a thorough bibliographic search (between 2017 and 2023) on pESI-positive *S*. Infantis genomic studies using a combined short- and long-read sequencing approach, 31 pESI-like sequences, obtained from public databases (National Center for Biotechnology Information [NCBI], accessed in May 2023), were selected and included in the analysis for comparison purposes (Table S1). The analysis was performed for all pESI-like sequences as follows: after annotation with Bakta v1.5.0 [25] (database updated on 11 November 2022), all pangenomes were analysed using Panaroo v1.3.3 (--clean-mode strict --aligner mafft --core_threshold 0.98) [26]. The threshold was set to 98%, and the percentage of length difference was set to 98%. The obtained results, including the clustering, were visualized using the Phandango tool [27].

### Alignment, phylogeny and pESI synteny

Before alignment, all circular sequences were rotated and linearized, starting with the origin of replication using the rotate script [28]. SNP analysis, without reference, was performed as follows: the pESI-like sequences were aligned using Mafft [29] with the FFT-NS-2 strategy, auto-selected by the tool. The final alignment in fasta format was used as input for the Gubbins tool v2.4.1 [30] to analyse the presence of variants/recombinations and phylogenetic relationships using the RAxML v8 tree builder [31]. The nucleotide substitution model GTRCAT for the first approximation and the nucleotide substitution model GTRGAMMA for the final phylogenetic reconstruction were used as suggested by the Gubbins tool, being the most suitable for our population, with default parameters. The tool did not take identical sequences into account in the final output; consequently, three pESI-like sequences were automatically removed from the analysis.

The obtained annotation and clustering of pESI-like were used as input for the linear visualization and study of the synteny by using gggenomes (https://thackl.github.io/gggenomes/articles/gggenomes.html) and ggtree [32] in R v4.3.0 core [33](www.R-project.org/).

## Results

### Genomic characterization of *S*. Infantis WGSs collection and phylogenetic tree

Analysis of the WGS data showed that, out of 232 sequences, 167 carried IncFIB(pN55391), *tet*(A) and *sul1* genes, and 65 lacked these genes; accordingly, the preferentially selected criteria. All the 232 *S*. Infantis isolates belonged to sequence type (ST) 32, but 1, a single locus variant of ST32, belonged to ST5275, was of animal origin (Italy) and displayed a different *sucA* allele (Table S1). IncFIB(pN55391) replicon was harboured by 167 isolates; of these, 162 harboured the *tet*(A) gene and all but one harboured the *sul1* gene. The *tet*(A) gene was present in three isolates, lacking IncFIB(pN55391) replicon, but of these, two isolates showed other pESI backbone markers (*K88*, *fim*, oriV_incP and pESI_backbone_gene) (Table S1). Thirty-nine *S*. Infantis isolates harboured the *bla*_CTX-M-1_ gene, of which all but one had the IncFIB(pN55391), but showed other pESI backbone markers (Table S1). Thirteen isolates carried the *bla*_CTX-M-65_ gene, all carrying the IncFIB(pN55391) replicon but lacking all pESI backbone markers (Table S1). All *bla*_CTX-M-1_- and *bla*_CTX-M-65_-positive isolates also carried *tet*(A) and *sul1* (Table S1).

SNP analysis of the 232 *S*. Infantis isolates showed between 1 and 2,074 SNPs compared to LN649235.1 reference, and 7 clusters (cluster I to cluster VII) were identified in the chromosomal SNP phylogenetic tree (Fig. 2). Of the seven clusters, six were based on a bootstrap value greater than 0.75 (all but cluster II, in which the subclusters presented a bootstrap value greater than 0.75), with two of those clusters originating from the root, whereas the other five shared a common ancestor (Fig. 2). The presence of pESI markers was identified in six clusters (Fig. 2). The clusters I–III, composed of 38 *S*. Infantis isolated from human, animal and food samples in Italy, 4 in the Netherlands and 2 in Belgium; all isolates, except 3, carried the *bla*_CTX-M-1_ gene. Cluster VI was composed of 16 isolates, mainly showing the *bla*_CTX-M-65_ gene. This gene was present in 1 isolate from food (Portugal) and 12 isolates from humans collected in Belgium, the Netherlands, Italy and Sweden. Cluster V contained 68 isolates, showing mainly *sul1* and *tet*(A) genes, from Poland and Portugal, which were countries that supplied only animal and food isolates. Cluster VII was characterized by the absence of pESI markers and resistance genes of pESI-like variable region, suggesting that the isolates lacking the pESI-like plasmid are grouped in the same cluster and are not scattered along the tree (Fig. 2, https://microreact.org/project/sinfantis-alba). This clusterization suggests that isolates with and without pESI-like plasmid have different chromosomal configurations.

### pESI pairwise distance and pangenome analysis

Forty-eight isolates, equally distributed in the six identified clusters with pESI markers, were selected for long-read sequencing (Table S1). The average size of the 48 fully resolved plasmids was 287,814 bp with a minimum size of 250,171 bp and a maximum of 325,457 bp (Table S1). The 31 complete sequences of pESI-like plasmids from different countries downloaded from the public database (accession numbers in Table S1) were added to the analysis.

**Fig. 3.**
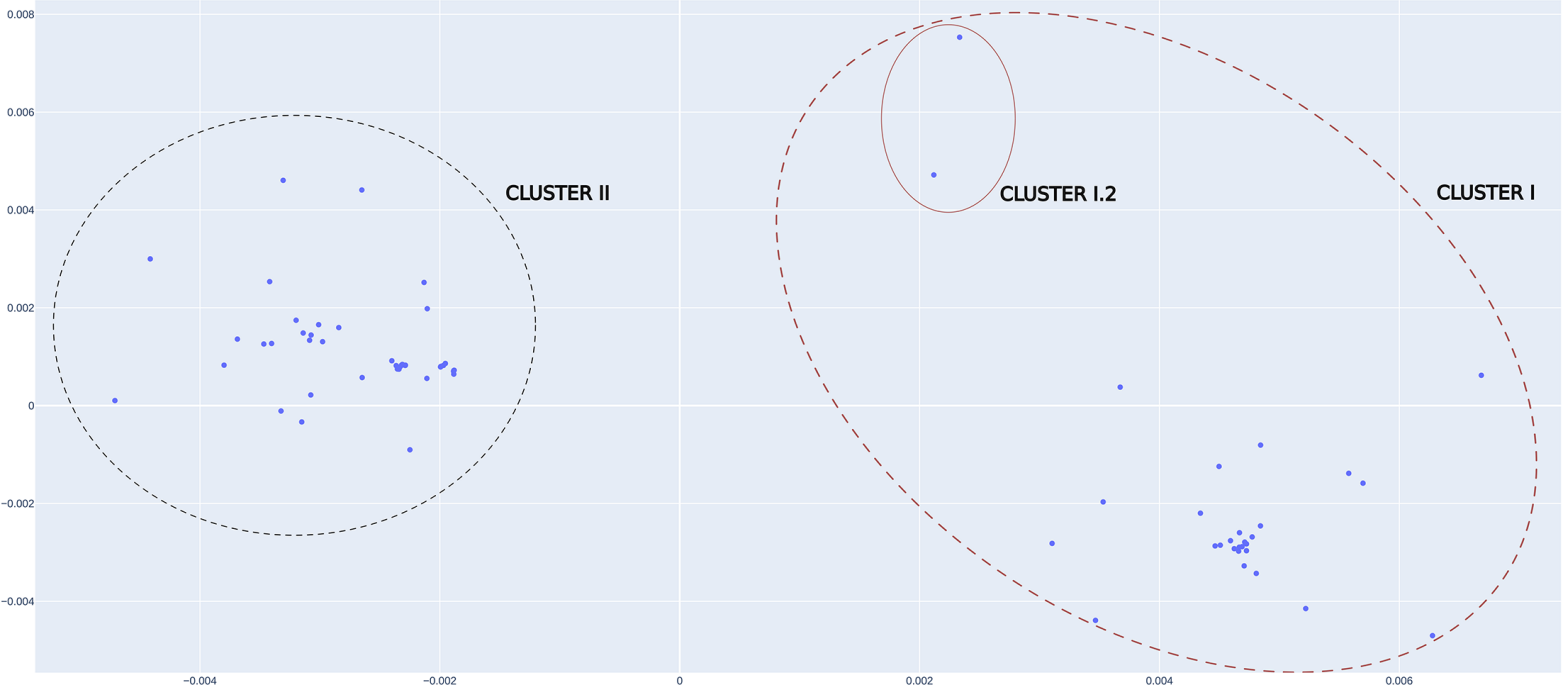
MDS plot built from mash triangle comparison of all complete megaplasmid sequences analysed. Sequences at right of the plot form the cluster I (including *bla*_CTX-M-65_-positive pESI sequences) with two ‘outlayers’ forming cluster I.2 at top right, including two pESI, one *bla*_CTX-M-14_-positive pESI. Sequences at the left of the plot form cluster II (including *bla*_CTX-M-1_-positive pESI sequences).

In the MDS plot obtained from the analysis of the 48 pESI-like closed plasmids and the 31 plasmids downloaded from NCBI, 3 separated clusters were observed based on the pairwise distance of the whole plasmid DNA sequences (Fig. 3). Cluster I included *bla*_CTX-M-65_-positive pESI-like sequences and the sub-cluster I.2 with only two *bla*_CTX-M-14_-positive pESI-like from Russia and a *bla*_CTX-M_-negative pESI-like from Norway (animal origin). Cluster II included *bla*_CTX-M-1_-positive pESI-like sequences.

According to the gene-by-gene analysis, 590 unique genes were identified in the 79 (48 fully sequenced plasmids plus 31 pESI plasmid sequences obtained from NCBI) pESI-like plasmids studied (Table S1). Of those, 148 coding sequences (CDSs) (covering around 50% of the megaplasmid length) were identified as core genes of pESI-like, as these CDSs were present in 99–100% of the plasmids analysed. Moreover, 74 additional genes could be considered as part of the soft-core genes because they were detected in 96–99% of the megaplasmids (Table S1).

The core genes included the replication gene *repA*, the partitioning encoding genes *parA*, *parB* and *sopA*; the genes involved in conjugation encoding for the Tra and the Pil families’ proteins comprising the shufflon system; the yersiniabactin operon; the nickel resistance operon; genes encoding fimbrial proteins including K88 and Fim; and toxin/antitoxin systems including CcdA/B or PemK/I (MazF/E) (Table S1). Other CDSs detected were DEAD/DEAH box helicase, HAAAP family transport protein and virulence-associated protein VagC (Table).

**Fig. 4.**
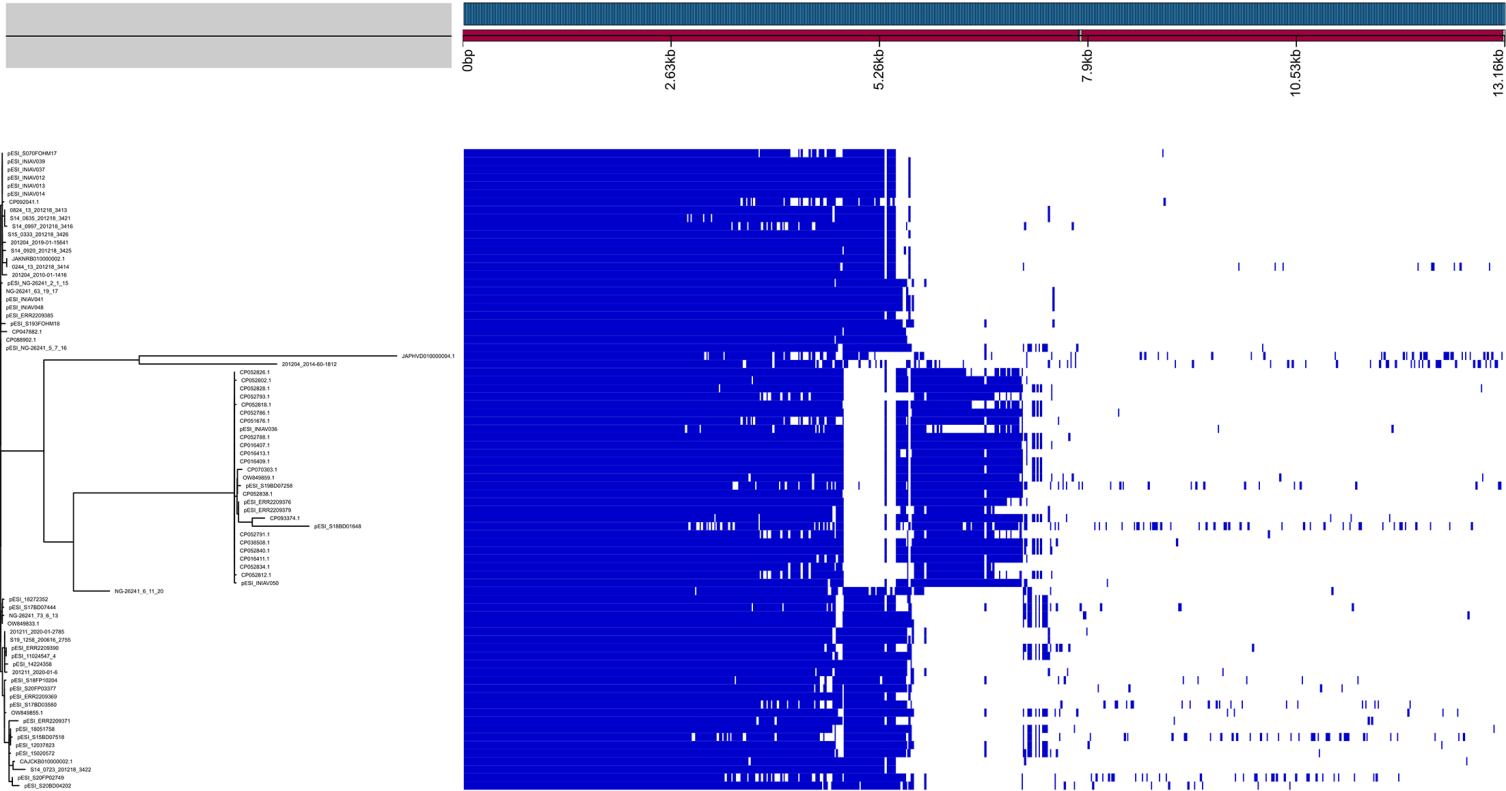
Clusterization of the pESI plasmids obtained from gene-by-gene analysis. The right part of the figure represents the presence/absence of the CDSs of pESI, being full blue when present and white when absent.

When comparing the CDS content of the pangenome in the obtained tree (Fig. 4), two main clusters (CTX-M-1 and CTX-M-65 clusters) emerged based on the accessory genes of the plasmid. Among these, a clear distinct group of genes was missing from the central plasmid cluster, all plasmids containing *bla*_CTX-M-65_, while they were present in the other pESI-like plasmids. One example is the chaperonin gene *groEL* (a heat shock protein), which was located in a *bla*_CTX-M-65_-positive pESI variant, without the gene encoding the co-chaperonin GroES. These chaperonins (both genes) have not been identified in the other pESI plasmid variants. Another example was the *ydgA* gene, encoding a protein involved in a signal process [34].

Similarly, a group of CDSs located in the central CTX-M-65 cluster was not detected in the CTX-M-1 cluster (Fig. 4). Notably, those coding genes included an extra copy of the toxin/antitoxin system VapBC family, the arsenic resistance operon, the *fip* (fertility inhibition of IncP plasmids) and *finQ* (transfer inhibition of IncF plasmids, without *finW*) genes.

### Phylogeny of pESI plasmid

**Fig. 5.**
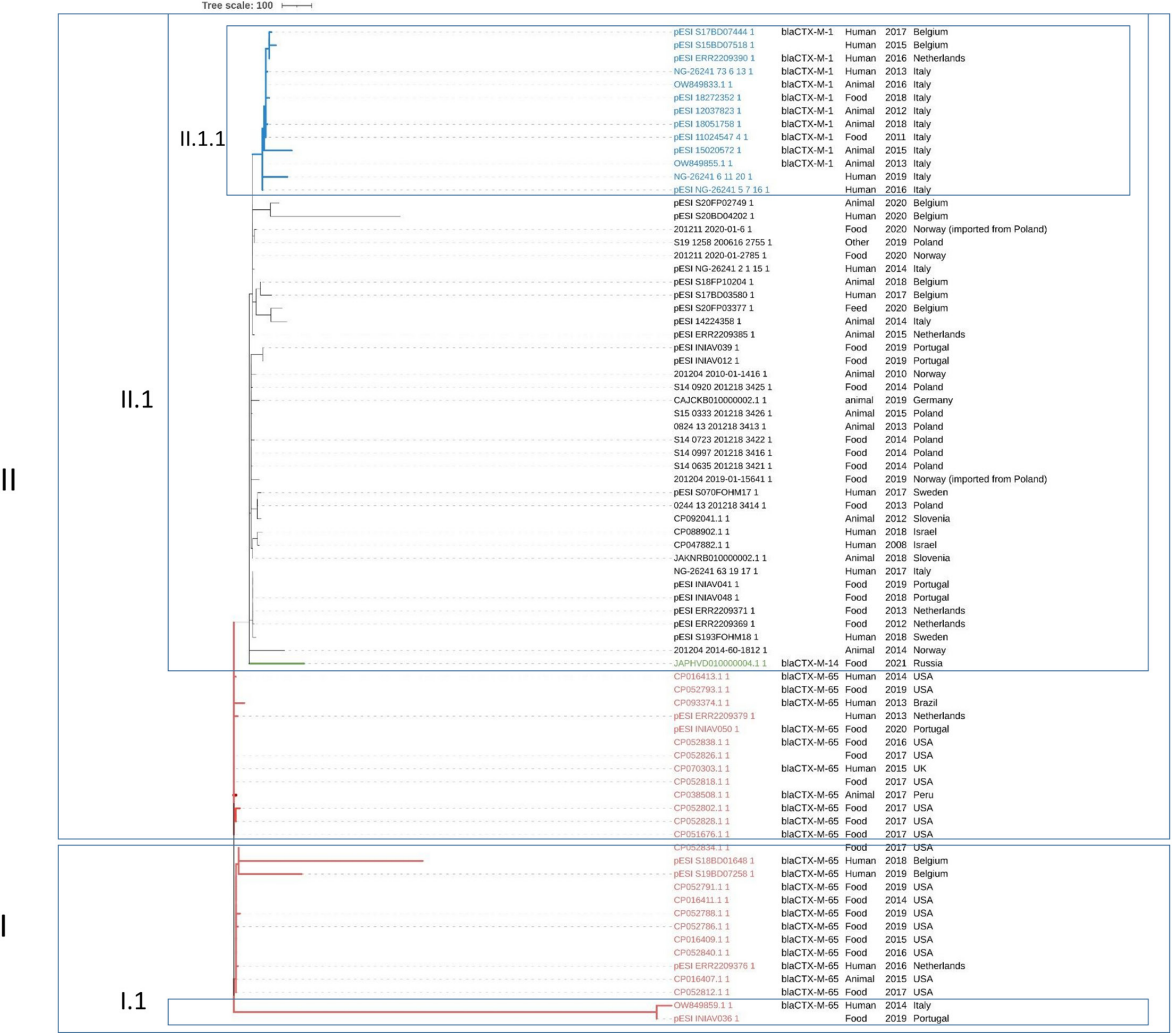
Phylogeny of pESI plasmids, built with the SNP approach from Mafft multi-alignment. In blue are marked the plasmids included in the subcluster II.1.1, including the *bla*_CTX-M-1_ pESI. In red are marked the pESI containing *bla*_CTX-M-65_ and genetically related pESI (without *bla*_CTX-M-65_), which are distributed between the Cluster I and Cluster II. In green is marked the pESI harbouring *bla*_CTX-M-14_.

A phylogenetic tree was constructed to study the differences based on SNPs and the effect of recombination events. In general, low genetic variability within the clusters was observed, despite the SNP distances (from 1 to 1,731 SNPs, according to the pairwise distance based on the polymorphic sites) or the recombination block (from 0 to 45). As shown in Fig. 5, the calculated branch lengths were short, with a few exceptions.

Using the SNPs approach, two main clusters were identified (Fig. 5); cluster I included 14 pESI-like plasmids, of which 12 were *bla*_CTX-M-65_ positive. Four of these plasmids, two from Belgium, one from the Netherlands and one from Portugal, were part of the study. They clustered together with pESI-like plasmid sequences obtained from NCBI, isolated from the USA and one from Italy (with travel to the USA) [4,6, 8]. Ten out of 12 pESI-like presented from 1 to 5 SNP differences, whereas the other 2 differed from 12 to 19 SNPs. Inside cluster I, there was a small subcluster I.1, containing two genetically distant pESI with >300 SNPs (Table S1). It was composed of two plasmids differing only in four SNPs, one from a food sampled from Portugal (pESI_INIAV036) and one Italian human clinical *bla*_CTX-M-65_-positive isolate (OW849859.1) from a patient with travel history to the USA (Table S1) [6].

The remaining 62 pESI-like plasmids from this study were grouped in cluster II, and within this cluster, a large subcluster, denominated II.1, including 49 pESI-like plasmids, was identified; these plasmids were from all European countries involved in this study, including Russia and Israel, and detected in *S*. Infantis isolated from food, feed, human and animal sources. Within this subcluster, genetically distant pESI-like sequences were noted: the Russian *bla*_CTX-M-14_-positive pESI-like JAPHVD010000004.1 (at least 10 SNP differences from all the other plasmids), the Norwegian pESI 201204_2014-60-1812 (at least 8 SNP differences with the other pESI plasmids from the cluster) and the pESI_S20BD04202 from Belgium (human) that was also genetically different (at least 24 SNPs of difference) (Fig. 5, Table S1). A subcluster within II.1, named II.1.1, contained 13 pESI-like (with 1 to 20 SNPs differences), 10 out of 13 were *bla*_CTX-M-1_ positives, from Belgium, Italy and the Netherlands, detected in *S*. Infantis isolated from food, human and animal samples (Fig. 5, Table S1).

Interspersed in cluster II, there were an additional 13 pESI-like. Ten out of 13 harboured *bla*_CTX-M-65_. Among these plasmids, the most divergent was the pESI-like from a *S*. Infantis isolated in Brazil, which presented more than five SNPs of difference from the rest of the pESI-like plasmids (Fig. 5).

### pESI synteny analysis

**Fig. 6.**
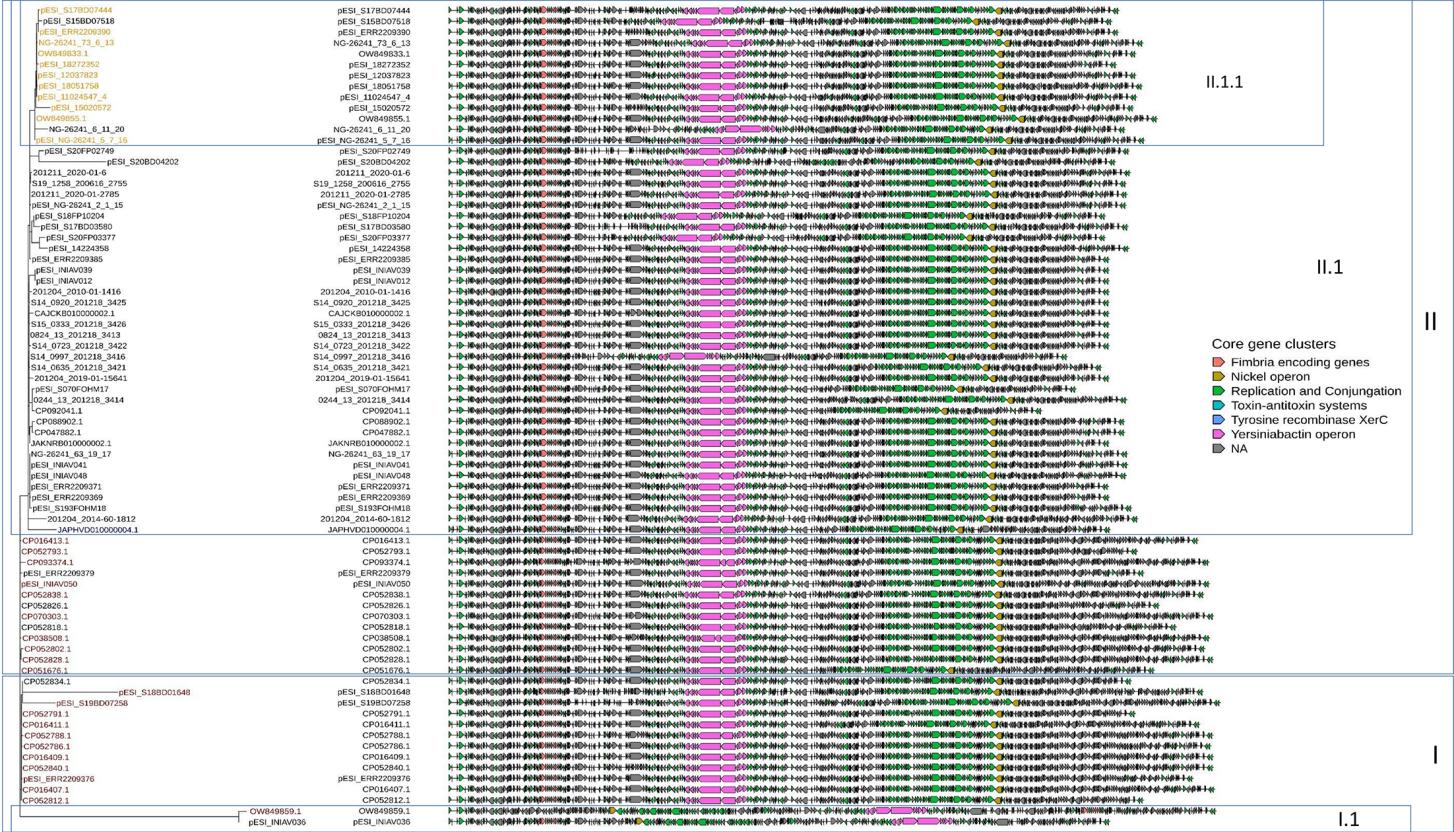
Synteny analysis, representing the entire length of the plasmid and CDS identified. Clusterization is based on the SNP tree. Yellow pESI labels indicated that pESI harbours *bla*_CTX-M-1_, red plasmid labels harbour *bla*_CTX-M-65_, and the blue plasmid labels harbour *bla*_CTX-M-14_. Some conserved CDS are coloured as indicated in the figure legend.

**Fig. 7.**
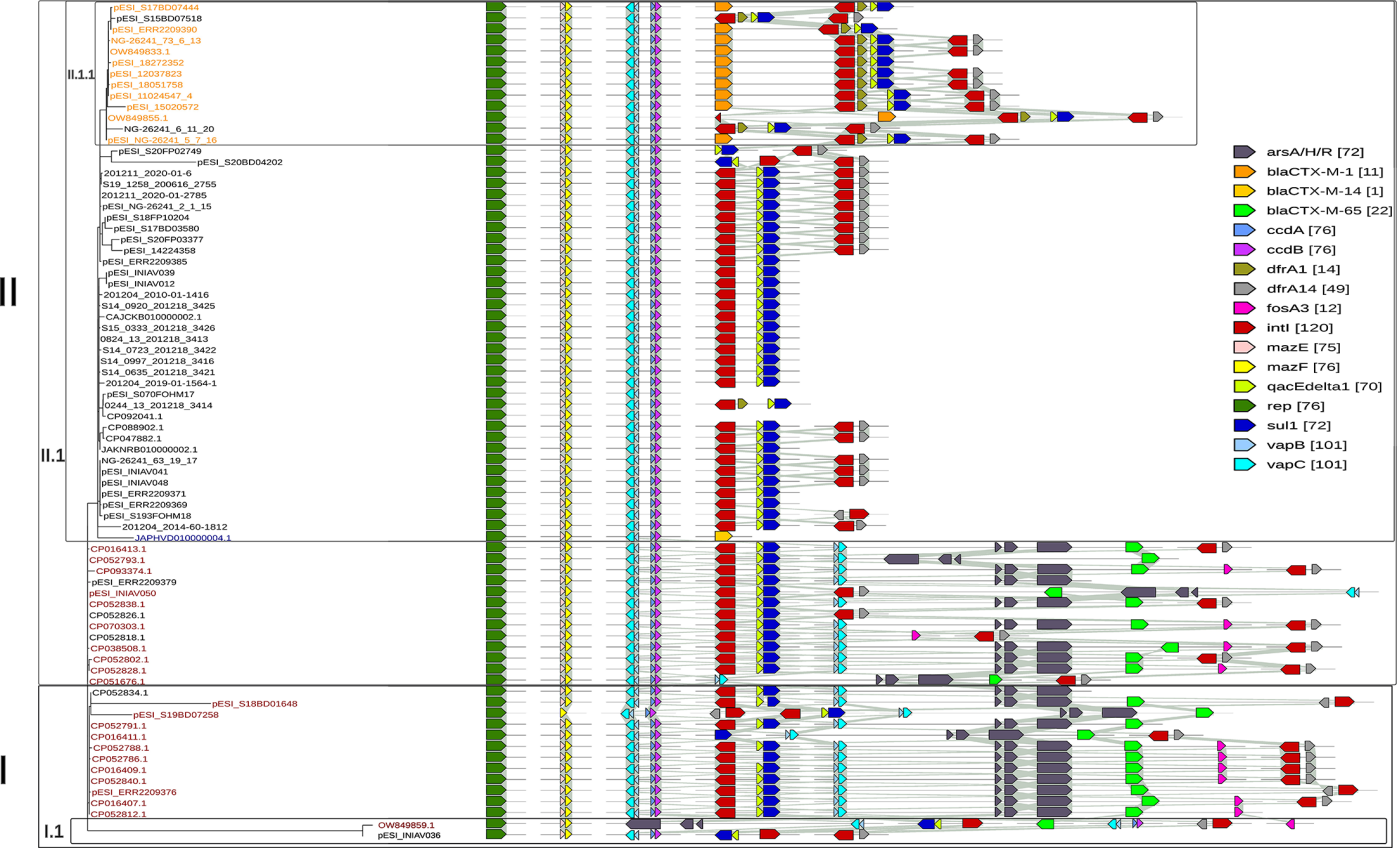
Synteny analysis, which includes the accessory genes and the origin of replication (*rep*). Regions containing conserved genes have been hidden. The accessory genes represented in the figure with a coloured arrow are listed in the legend. Clusterization is based on the SNP tree. Yellow pESI labels indicated that pESI harbours *bla*_CTX-M-1_, red plasmid labels harbour *bla*_CTX-M-65_, and the blue plasmid labels harbour *bla*_CTX-M-14_.

The analysis of plasmid synteny indicated a high stability in the pESI-like structure, as shown in Fig. 6. The position of core genes was conserved in the plasmid, besides the insertion/deletion of variable genes. The *bla*_CTX-M-1_ and the *bla*_CTX-M-65_ pESI-like plasmids were easily identified as part of different clusters and subclusters. However, when analysing the position of accessory genes in the phylogeny, significant differences were observed regarding the relative gene positions (Fig. 7). The most relevant difference is the localization of the *bla*_CTX-M_ genes: the *bla*_CTX-M-1_ gene was located upstream the integron containing the *dfrA1* and *sul1* genes cassette, whereas *bla*_CTX-M-65_ was located between *ars* operon and the second integron containing the *dfrA14* gene cassette (Fig. 7). The different plasmid structures almost fit within the identified clusters by SNPs (Fig. 5), but not completely. For some plasmids, such as OW849859 and INIAV036, the differences were evident because of the arrangements. However, some plasmids, such as CP016413 and CP052793 with different rearrangements (the presence of *intI2* and *dfrA14* genes in CP016413, inversion of the *ars* operon in CP052793), were not distant from each other (two SNPs of difference) (Fig. 7, Table S1). In subcluster II.1.1, where all the *bla*_CTX-M-1_-positive pESI-like plasmids were located, the observed variability seemed due mainly to the presence of one or two integrase genes and their relative positions.

The genetic composition of the variable regions remained stable (Fig. 7). For example, in the *bla*_CTX-M-1_-positive pESI-like, the integron 1 harboured *dfrA1*, *qac*EΔ1 and *sul1*, whereas the *bla*_CTX-M-65_ gene was always present downstream of the arsenic operon and the *fosA3* gene in 54.5% of the *bla*_CTX-M-65_-positive plasmids studied (Fig. 7).

## Discussion

Studying the biology and evolution of plasmids is fundamental to understanding the transmission of the AMR determinants through horizontal gene transfer events in foodborne zoonotic pathogens. In the present study, we analysed in depth a WGS collection of *S*. Infantis from human, animal and food from seven countries. A hybrid approach of short and long reads was used to assemble and characterize the pESI-like megaplasmid, responsible for MDR in one of the most prevalent *Salmonella* serovars. Two main types of pESI variant plasmids found in *S*. Infantis have been described, with the main difference being the accessory gene content, including the *bla*_CTX-M_ variant involved (Figs 3 and 4). As previously reported in the literature, the presence of *bla*_CTX-M-1_ in pESI-like has been related to European *S*. Infantis and reported mainly in Italy [4,6], whereas the *bla*_CTX-M-65_ has been mainly described in South and North America [7,9, 15, 35].

The pESI-like structure, including the composition of core genes and the presence of recombination and integration events, as well as its microevolution over the years in *S*. Infantis using a time-reversible model, has been deeply studied in this work. Our results indicated a certain stability of three pESI-like variants (harbouring, respectively, *bla*_CTX-M-1_ or no *bla*_ESBL_ genes and *bla*_CTX-M-65_) detected in *S*. Infantis from different geographical regions and sources, over time (2011–2021) (Figs 3 and 5). Moreover, it has also been proven that 50% of the megaplasmid genetic content was the same in all the pESI-like sequences, confirming that the differences were mainly related to the acquisition of resistance, virulence or fitness-enhancing genes that would potentially benefit the *Salmonella* host. A substantial agreement was observed between the phylogenetic tree of pESI-like plasmids (Fig. 5), built considering also recombination gaps, and the pairwise distance analysis (Fig. 3). In particular, cluster II of the MDS representation includes all plasmids grouped in subclusters II. and II.1.1 of the phylogenetic analysis, except the two most distant from Norway and Russia. Cluster I includes the isolates from cluster I and the subclusters from cluster II of the SNPs. Moreover, the phylogenetic tree of pESI-like plasmids agreed with the clustering based on the pangenome (Fig. 4) and with the synteny analysis (Figs 6 and 7).

It has been observed that some virulence genes, such as the aerobactin operon, are conserved in pESI-like, being included among the pESI core genes [6]. Other virulence genes classified as core genes are two toxin/antitoxin systems (*pemI/K*) and *vapBC*: they seemed to be a characteristic of the *bla*_CTX-M-65_-positive pESI variant because in those isolates the toxin/antitoxin system was duplicated, which contributes to plasmid maintenance during vertical transmission and could contribute to the pESI stability [36].

The low genetic distances in the phylogenetic analysis may be due to the fact that pESI-like is a relatively recent plasmid, hypotetized to have emerged in the late 1980s in European countries by Guzinsky *et al*. in a time-measured BEAST phylogeny [35]. Moreover, it has been hypothesized that the emergent *S*. Infantis (ESI)-CTX-M-65 cluster originated in South America, then became dominant in poultry sources in North America and, favoured by the pESI variant, spread through clonal expansion [15,37, 38]. The differences observed between the pESI clusters are mainly due to the incorporation of new genes, and in some cases, to rearrangements, irrespective of the source of isolation or country. Some pESI-like plasmids from Belgium showed a tendency to accumulate mutations that would lead to a greater differentiation, as indicated by the elevated number of SNPs in comparison with pESI-like plasmids isolated from other countries.

The clustering obtained from gene-by-gene analysis (Fig. 4) and the phylogeny of pESI-like plasmids, built with SNP approach from mafft multi-alignment (Fig. 5), agrees with the chromosome-based *S*. Infantis SNP phylogenetic tree (Fig. 2). This indicates high stability of pESI-like and agreement between isolates clustering and pESI plasmidic variants. However, this conclusion is in contrast with the earlier observation in Alba *et al.*, based on the analysis of only short-read sequences and isolates collected in a smaller timeframe [6]. The discrepancies may be attributed to the more complete analysis enabled by resolved plasmid sequences obtained through ONT technology.

One of the main difficulties in the phylogenetic analysis was the calculation of time substitution and the model of substitution itself, so for the scope of the study, we considered that the mutation rate of the megaplasmid was similar to the mutation rate of the chromosome. This approximation is based on the length of the plasmid and, therefore, the assumption that few copies (even only one) would coexist in the host and on the relatively recent origin of the plasmid, but it may have influenced the results about the high stability of the pESI-like plasmid.

A recent study found that ~66% of *Salmonella* plasmids are mosaic plasmids [39], a feature that is also found in the pESI-like megaplasmid. The diversity of AMR and virulence genes in pESI-like is probably associated with the selection pressure from different antibiotics and/or heavy metals, contributing to the success of such an MDR and sometimes ESBL-producing lineages through the complex mechanism of co-selection (i.e. tetracyclines, trimethoprim, sulphonamides and aminoglycosides; see the genetic basis of such an MDR profile within the plasmid structures). These features could also be explained by the strong ability of this plasmid to acquire and incorporate resistance genes [6]. The differences observed, due to structural changes, mutation and acquisition of new genes, confirm the existence of two well-differentiated plasmid variants and the possible expansion of a new variant harbouring *bla*_CTX-M-14_.

In the *bla*_CTX-M-65_ variant, it has been confirmed that the *fosA3* gene is a marker of the *bla*_CTX-M-65_-positive pESI variant, as well as the resistance genes to arsenic (*ars* gene). These features prompt speculation on the acquisition of resistance to fosfomycin (e.g. through human use for Urinary Tract Infections [UTIs] against Enterobacterales) and a possible co-selection with *bla*_CTX-M-65_ and on selection pressure exerted by the presence of arsenic in the farm environment of primary productions [40]. The increase of resistance to fosfomycin is a cause for concern for human health because the lack of new antimicrobial agents has led to a reconsideration of older antibiotics, including fosfomycin, for treating MDR infections, which would prove ineffective [41]. Similarly, noteworthy is the presence of the *mer* operon (mercury resistance) in those plasmids, also described by Lee *et al.* [7]. The coexistence of metal-resistant genes and AMR genes on the same plasmid poses a threat to public health [7,42, 43].

Conversely, the structure of the CTX-M-1-pESI plasmid harboured more fitness-enhanced genes as ‘accessory’ genes. Some examples are a copy of *gro*EL, a heat chaperonin protein, and genes involved in the signalling, such as *ydgA*, that could have a role in the spread of this variant of pESI in Europe. Nevertheless, this type of megaplasmid conserved the resistance genes for four main antimicrobial classes, indicating that the co-selection phenomenon also led to the expansion of the plasmid in Europe.

Our study also found an elevated concordance and stability between the pESI type and the clones circulating in Europe and, therefore, related to the country and sources (human, animal and food) (Figs 2 and 5). Several previous studies on the phylogeny and evolution of *S*. Infantis have been carried out in different countries, such as several European countries, South Africa, the UK and the USA [6,15, 35, 37, 38, 44].

In particular, our results identified cluster V (food and animal) with pESI-like, but lacking the ESBL gene, and cluster VII, mainly composed of *S*. Infantis isolated in Sweden and Norway, lacking the pESI-like plasmid and the ESBL gene, as the largest (Fig. 2). Interestingly, *S*. Infantis isolated from animal and food in Italy and Sweden did not appear in cluster V. Italian isolates from animal and food were mainly distributed along clusters I–III, which are composed of *bla*_CTX-M-1_ carrying isolates, and human-derived strains, and for Swedish isolates in cluster VII, which is pESI-like negative. Additionally, the human and food/animal isolates from Belgium, lacking the ESBL genes, shared the same branch of cluster IV, indicating the presence of the same clone in both sources. This confirms, in agreement with previous observations [4,6, 8], that *S*. Infantis isolates from Italy, sharing the same chromosomal structure, can circulate in animals, meat (food) and humans.

Overall, in agreement with the most recent EU Summary Reports on AMR [1], we found that in animal and food, CTX-M-1 was the dominant variant (13.8%), mainly from Italy. In any case, two *bla*_CTX-M-1_-positive isolates from the Netherlands (broiler meat products) investigated in our study confirm that these genetic features have been spreading throughout Europe across different sectors. These data are consistent with the low levels of extended-spectrum cephalosporin resistance reported in Dutch flocks [1], indicating that this resistance has spread in *S*. Infantis in the Netherlands. CTX-M-65 remains a very sporadic feature in the European animal isolates (0.7%), with no isolate from poultry, and only one (*n*=1) from a food sample of meat (unknown origin) in Portugal. While in human isolates, the proportion of *bla*_CTX-M-1_ and *bla*_CTX-M-65_ positive was 21.8% and 17.8%, respectively.

This work represents a comprehensive study about the structure and composition of this relevant plasmid of *S*. Infantis, also confirming the circulation of the pESI-like harbouring *bla*_CTX-M-1_ in the ESBL-producing *S*. Infantis in Europe and highlighting the importance of the implementation of plasmid sequencing for Public Health surveillance purposes in a One Health perspective. Notably, surveillance systems relying exclusively on short-read sequences fail in resolving plasmid structures and monitoring emergent high-risk plasmids containing multiple resistance genes. The implementation of plasmid sequencing through long-read technologies represented the main goal of the Full-Force Project (https://onehealthejp.eu/projects/antimicrobial-resistance/jrp-full-force) and other EU initiatives, which aimed at improving epidemiological surveillance of Mobile Genetic Element (MGE)-mediating AMR by promoting capacity building in plasmid sequencing across major Public Health Institutes, conducting proficiency tests and strengthening shared data standards for MGE analysis. By providing more comprehensive and complete genomic information, long-read sequencing offers a robust framework for studying the evolution and spread of AMR through both clonal expansion and horizontal gene transfer in a One Health context. The use of advanced sequencing technology would permit tracing the plasmid spread and the clonal expansion of zoonotic pathogens through the veterinary, food, environment and human sectors [45,46].

## Supplementary material

10.1099/mgen.0.001787 Supplementary Material 1.

## Abbreviations

MGE: Mobile Genetic Element
NCBI: National Center for Biotechnology Information
UTIs: Urinary Tract Infections
